# Longitudinal profiles of occupational physical activity during late midlife and their association with functional limitations at old age: a multi-cohort study

**DOI:** 10.1007/s00420-023-02003-5

**Published:** 2023-07-29

**Authors:** Kyrönlahti Saila, Nygård Clas-Håkan, K. C. Prakash, Neupane Subas

**Affiliations:** 1 Faculty of Social Sciences, Unit of Health Sciences, Tampere University, Tampere, Finland; 2 Gerontology Research Center, Tampere University, Tampere, Finland

**Keywords:** Ageing workers, Biomechanical exposure, Functional ability, Physical activity paradox, Trajectory analyses

## Abstract

**Objective:**

The aim was to examine longitudinal profiles of occupational physical activity (OPA) from midlife to retirement and to investigate how the different OPA-profiles are associated with mobility limitations (ML) and activities of daily living (ADL-disability) at old age.

**Methods:**

Harmonized data from two cohort studies from Finland and the United States, that have followed people from midlife until old age were used. Repeated measurements of self-reported OPA were collected during approximately 11- to 12-year period. Persons who had data on OPA from ≥ 2 time points during the period from mid-working life to retirement were included. Latent class growth analysis was used to identify OPA-profiles. Risk ratios (RRs) with 95% confidence intervals for the associations of the OPA-profiles and later life MLs and ADL-disability were estimated.

**Results:**

Three OPA-profiles were identified in both cohorts: high-persistent, moderate-fluctuating, and low-persistent. For majority OPA remained stable: for workers reporting high or low levels of OPA at midlife, the physical demands of work likely persisted, whereas people reporting moderate level OPA had high fluctuation in their exposure level. Members of high-persistent and moderate-fluctuating OPA-profiles had increased risk of subsequent MLs at old age. The RRs for ADL-disability did not differ between the profiles.

**Conclusions:**

Perceived OPA remains rather stable for workers reporting high or low physical work demands during midlife, yet fluctuating profiles also exist. Workers exposed to high or moderate OPA have higher risk for MLs when they reach old age. Establishing safe and health-promoting levels of OPA during late working life might have positive long-term consequences on healthy ageing.

**Supplementary Information:**

The online version contains supplementary material available at 10.1007/s00420-023-02003-5.

## Background

With the aging of the population, healthy aging, “the process of developing and maintaining the functional ability that enables wellbeing in older age” (Beard et al. 2016), is one of the main public health priorities. According to the life-course theory, healthy aging is influenced by different experiences across the life course (Diana Kuh 2019). Work-related exposures in earlier life stages are established and potentially modifiable determinants of healthy aging, i.e., adverse working conditions inhibit whereas good working conditions facilitate healthy aging (Diana Kuh et al. 2014). Aging workers may be particularly susceptible to adverse physical work exposures as with age, the body’s systems become less able to repair damage caused by accumulated exposures resulting in progressively declining physical capacity approximately from the fifth or sixth decade of life on (Diana Kuh et al. 2014). Adverse exposures during this sensitive period may have long-term consequences on functional ability beyond working life.

Occupational physical activity (OPA) is an important determinant of health and functional ability. While leisure-time physical activity (LTPA) is well-evidenced to improve health (World Health Organization 2020), a high level of OPA is on the contrary associated with detrimental health outcomes, such as cardiovascular disease (Holtermann et al. 2021), functional limitations (Aittomäki et al. 2005; Hinrichs et al. 2014; Leino-Arjas et al. 2004; Mänty et al. 2014; Prakash et al. 2016; 2017; Sabbath et al. 2013) and all-cause mortality (Coenen et al. 2018; Holtermann et al. 2021). Moreover, high OPA is strongly associated with adverse work outcomes such as declining workability, and preterm exit from the workforce (Sterud 2014; Sundstrup et al. 2018). Despite considerable evidence of the adverse effects of OPA, the two modalities of physical activity are not differentiated in the official WHO physical activity recommendations (World Health Organization 2020). Instead, the recommended amount of physical activity can be accumulated during leisure time or as part of one's work. OPA is often characterized by long duration, static and constrained postures and activities as well as insufficient recovery time (Holtermann et al. 2018). Instead of producing health benefits, OPA may in long-term cause bodily impairments, such as musculoskeletal disorders (Nygaard et al. 2022). According to the disablement process the accumulation of bodily impairments may eventually lead to functional limitations and disability (Verbrugge and Jette 1994). Thus, repeated occupational physical strain may cause maladaptive bodily responses leading to differential disability processes for workers with different OPA profiles.

Previously, researchers have provided evidence of the long-term consequences of OPA beyond retirement, which suggest that physically demanding work during midlife predicts functional limitations later in life (Hinrichs et al. 2014; Mc Carthy et al. 2013; Prakash et al. 2016; 2017; Russo et al. 2006; Rydwik et al. 2013). In the previous studies, however, exposure to OPA was measured on the basis of a single baseline assessment (Hinrichs et al. 2014; Leino-Arjas et al. 2004; Prakash et al. 2016; 2017), or either occupation-level or rétrospective exposure estimates were used (Mc Carthy et al. 2013; Russo et al. 2006; Rydwik et al. 2013), which do not reliably consider the variation across time (Andrasfay, Pebley, and Goldman 2021). Cumulative exposure to perceived OPA, determined by several repeated assessments, could potentially inform more about the development of the exposure level with age as well as about heterogeneity between individuals. However, there is lack of prospective studies, in which a person-centered investigation strategy was used.

In the current study, using two prospective cohorts from Finland and the USA, we first investigate the developmental patterns of perceived OPA among ageing workers. Secondly, we investigate the association of the different profiles of OPA with mobility limitations and disability.

## Methods

### Study population

Harmonized data from two independent cohort studies from Finland, and the USA in which people from mid-working life to old age have been followed, were used to examine the OPA profiles and their association with old age functional limitations. These cohort studies included data on the main exposure, OPA covering approximately an 11-to 12-year period as well as pertinent covariate and outcome data.

Finnish Longitudinal Study on Ageing Municipal Employees (FLAME) is a 28-year follow-up study of Finnish municipality workers, conducted by the Finnish Institute of Occupational Health from 1981 to 2009 (Hinrichs et al. 2014; Ilmarinen et al. 1991). In 1981, a postal questionnaire was sent to 7344 municipal workers, aged 44–58 years, working in all municipal professions in Finland. A total of 6257 (85.2%) persons responded to the baseline questionnaire. Follow-up data were collected in 1985, 1992, 1997 and 2009. To be eligible for the analytical sample used in the trajectory analysis, the participants needed to have answered to the questionnaire related to OPA in the baseline survey and at least one of the proceeding surveys conducted in 1985 or in 1992 (n = 5108) (Figure S1).

Health and retirement study (HRS) is an ongoing, open access, longitudinal panel study of representative sample of Americans over the age of 50 years, conducted by the University of Michigan (Sonnega et al. 2014). The data is collected biennially through telephone interviews. A baseline cohort with 12652 respondents was launched in 1992. As an analytical sample for the trajectory analysis, we included respondents who had data on OPA at baseline and from at least one of the follow-ups from the biennial survey waves between 1992 and 2004 (n = 4161) (Figure S1).

For the regression analyses, the eligibility criteria were further restricted to those who had information on the study outcomes; mobility limitations (n = 2544 in FLAME, and n = 2126 in HRS) and disability (n = 2596 in FLAME, and n = 1299 in HRS). The outcomes were assessed 28 years after the baseline in FLAME (in 2009) and 24 years after the baseline in HRS (in 2016). During the follow-up approximately a third of the study sample died in both cohorts (n = 1560 in FLAME, n = 1260 in HRS), whereas 19% in FLAME (n = 952) and 38% in HRS (n = 1602) were dropped out. Those who either had information available on disability or died during the follow-up were included in competing risk analyses (n = 4156 in FLAME, n = 2559 in HRS). Mortality data was obtained from the Finnish National population register for FLAME study participants. For HRS participants mortality information were obtained from a combination of two data sources: reports from household members and records retrieved through matching with the National Death Index.

FLAME was approved by the ethics committee of the Finnish Institute of Occupational Health (Helsinki, Finland), and the participants gave written informed consent. For the HRS Study, ethical approval was obtained from the University of Michigan Institutional Review Board. The interviewer read a confidentiality statement at a first contact and by agreeing to the interview respondents gave verbal consent.

### Exposure assessment

OPA was determined similarly in both cohorts at each wave, based on participants’ self-reports on current exposure to three different types of physical exposures: (1) heavy physical workload, (2) working in an awkward position and (3) carrying and lifting heavy objects. The original response options ranged from 0 to 4. For the analysis, each of the three OPA items were first categorized into low (response options 0 = not at all, and 1 = seldom), moderate (response option 2 = moderately) and high (response options 3 = often, and 4 = very often). Finally, a single three-category variable was created for OPA, and respondents were categorized based on their highest exposure: those who reported low in all three items were categorized into *the low* category, those who reported moderate in one or more items were categorized into *the moderate* category, whereas respondents reporting a high level for any of the items were categorized into *the high* category.

In FLAME the exposure assessment period was 11 years during which the same questions were asked three times (in 1981, 1985 and 1992). Respondents on average provided exposure data at 2.71 (SD 0.46) timepoints of the possible three study waves. In HRS the assessments period was 12 years (1992–2004). The included respondents provided exposure data on average, at 2.91 (SD 1.04) timepoints of the possible seven biennial study waves.

### Outcome assessment

Mobility limitations were assessed using two commonly used mobility items: (1) self-reported ability to climb several flights of stairs without resting, and (2) walk approximately 500 m (FLAME) or several blocks (HRS). The level of difficulty in these activities was asked. The answer options had four levels, ranging from not possible to no difficulty. The respondents were categorized into two categories: “no mobility limitations” comprising the respondents who reported being able to perform both tasks without any difficulties, and “mobility limitations” comprising respondents who reported being unable to perform the tasks or having at least some difficulties (Kyrönlahti et al. 2021).

Disability was assessed with items from a validated Activities of Daily Living (ADL) -questionnaire (Sidney 1983) in both cohorts. Five daily self-care activities were included: dressing, bathing, eating, getting in/out of bed, toileting. Respondents were categorized as “independent in ADL” (no difficulties in any of the tasks) or “dependent in one or more ADL” (any level of difficulty or unable to perform at least one of the ADL tasks).

### Assessment of covariates

Information on covariates was obtained from the baseline. Education was categorized in FLAME as low (primary school or less with no vocational training), intermediate (primary school combined with vocational training), and high (matriculation examination, or more). In HRS education was defined as low (high school at most), medium (high school graduate and some college), and high (college or more).

Other covariates included lifestyle factors (LTPA, smoking and alcohol consumption), body-mass index (BMI), and chronic conditions. To measure LTPA, participants were asked how often on average they exercised and strained themselves physically in their leisure time during the previous year based on which LTPA was classified as low (main activities do not involve moving/physical strain), moderate (some form of exercise ≤ 1 time/week) or high (brisk exercise ≥ 1 h/week or light exercise several times a week).

The participants were asked whether they have ever smoked cigarettes (more than 100 cigarettes during their lifetime) and whether they currently smoke. Based on their smoking behavior, the participants were categorized as never, former, and current smokers. Alcohol consumption was categorized as abstinent, moderate, or high based on self-reported frequency of consumption of alcoholic beverages (never, ≤ twice a month, ≥ once a week, in FLAME, and never, < daily, ≥ one drink/day in HRS, respectively.)

BMI (weight in kilograms per height in meters squared, kg/m^2^) was calculated from participants’ self-reported weight and height and categorized into three groups for the analysis (< 25 kg/m^2^, 25–30 kg/m^2^ and > 30 kg/m^2^). Participants’ self-reported, physician-diagnosed or -treated diseases, illnesses or injuries included respiratory disease, cardiovascular disease, diabetes, musculoskeletal diseases, and accidental injuries.

### Statistical analysis

Latent class growth analysis (LCGA) was used to study heterogeneity in the development of OPA among the respondents separately in both cohorts. Respondents were classified into OPA profiles based on individual exposure patterns to OPA during the follow-up. LCGA is a person-centered statistical method to identify and describe distinct subgroups of individuals that follow a similar pattern of change over time on a given feature (Muthén 2004). It allows the data to be summarized by a finite set of unique polynomial functions each corresponding to a discrete profile (Muthén 2004).

To identify the OPA profiles, we first fitted increasing number of trajectory models with a quadratic shape until the fit statistics showed no improvement in model fit. Models with 1–5 classes were evaluated. The best fitting solution was determined based on statistical model fitting criteria (Nylund, Asparouhov, and Muthén 2007), including Akaike Information Criterion (AIC), Bayesian Information Criterion (BIC), entropy, and the posterior class probabilities for most likely latent class membership (Supplementary Table 1). We also considered the size of the classes and interpretability of the results in choosing the optimum models. Next, we tested whether the fit of the chosen model improved with either linear or cubic shape (Nagin 1999). Mplus software was used for the LCGA.

The derived class assignment information was then used as an independent variable in a modified Poisson loglinear regression analyses in which the association between the profiles and the outcomes were examined adjusting for confounders at two phases: first model was adjusted for age and sex, and the second model was further adjusted for education, the lifestyle factors, BMI and morbidity*.* Interaction between OPA profiles and sex was not statistically significant, therefore results were not stratified by sex. Robust 95% confidence limits were calculated for the risk ratios (RR) using the robust standard errors. Death was deemed a competing risk for ADL-disability (Zajacova and Burgard 2013), therefore we repeated the same analyses using death as competing risk for ADL-disability. The competing outcome was defined as either having ADL-disability at the last follow-up or death during follow-up. We pooled the study-specific effect estimates and their standard errors in random-effects meta-analysis and assessed heterogeneity with the I^2^ statistic for both outcomes.

To assess the effect of sample attrition on the estimates, we conducted attrition analyses (Table S2). Differences between included and excluded (those who died during or were lost to follow-up) participants were assessed using chi-square test for categorical variables and ANOVA for continuous variables.

## Results

The baseline characteristics of the participants are presented in Table 1. The mean age of the study population was 50.3 (SD 3.6) years in FLAME and 55.4 (SD 3.8) years in HRS. The proportion of women was 56% and 47%, respectively. Slightly less than half of the participants had at least intermediate-level education. Average years of work seniority was approximately four years higher among FLAME participants than among HRS participants (19.6 vs. 15.9 years). The distribution of participants in occupational groups was similar in both cohorts (60% and 61% white collar workers in FLAME and in HRS, respectively). Majority were involved in high LTPA. Around 10% of the FLAME respondents were classified as obese (BMI > 30 kg/m^2^), which was lower than among HRS respondents (22%). The proportion of study participants in high alcohol consumption (10% in FLAME and 16% in HRS) and current smoking group (18% in FLAME and 24% in HRS) was lower than the other two groups.

**Table 1:** Baseline description of the study samples from the Finnish Longitudinal Study of Municipal Employees (FLAME), and the Health and Retirement Study (HRS)

	FLAME (n = 5108)	HRS (n = 4161)
Age, years, mean (SD)	50.30 (3.58)	55.44 (3.78)
Women (%)	2879 (56.4)	1938 (46.6)
Men (%)	2229 (43.6)	2223 (53.4)
Occupational group (%)		
White collar	3050 (59.7)	2574 (61.9)
Blue collar	2058 (40.3)	1585 (38.1)
Work seniority in current occupation, years, mean (SD)	19.64 (8.67)	15.88 (12.14)
Leisure-time physical activity (%)		
Low	1084 (21.2)	843 (20.3)
Moderate	1461 (28.6)	837 (20.1)
High	2471 (48.4)	2481 (59.6)
Education (%)		
High	534 (10.5)	1110 (26.8)
Intermediate	1943 (38.0)	742 (17.9)
Low	2599 (50.9)	2293 (55.3)
Body mass index		
< 25 kg/m^2^	2377 (47.0)	1489 (35.8)
25–30 kg/m^2^	2230 (44.1)	1770 (42.5)
> 30 kg/m^2^	454 (9.0)	899 (21.6)
Alcohol consumption, %		
Abstinent	3514 (69.2)	1418 (34.1)
Moderate	1043 (20.5)	2084 (50.1)
High	522 (10.3)	659 (15.8)
Smoking, %		
Never	2881 (56.4)	1566 (37.6)
Former	1312 (25.7)	1598 (38.4)
Current	914 (17.9)	997 (24.0)
Chronic conditions, %		
Respiratory disease	618 (12.1)	363 (8.7)
Cardiovascular disease	1096 (21.5)	390 (9.4)
Musculo-skeletal disease	1680 (32.9)	1366 (32.8)
Diabetes	115 (2.3)	294 (7.1)
Accidental injury	603 (11.8)	534 (12.8)

Summary statistics are calculated among participants with non-missing data; missing data in FLAME included work seniority (*n* = 22), leisure-time physical activity (*n* = 92), education (*n* = 32), body mass index (*n* = 47), alcohol consumption (*n* = 29), smoking (*n* = 1), chronic conditions (*n* = 1), and in HRS occupational group (*n* = 2), work seniority (*n* = 14), education (*n* = 16), body mass index (*n* = 3)

**Fig. 1:**
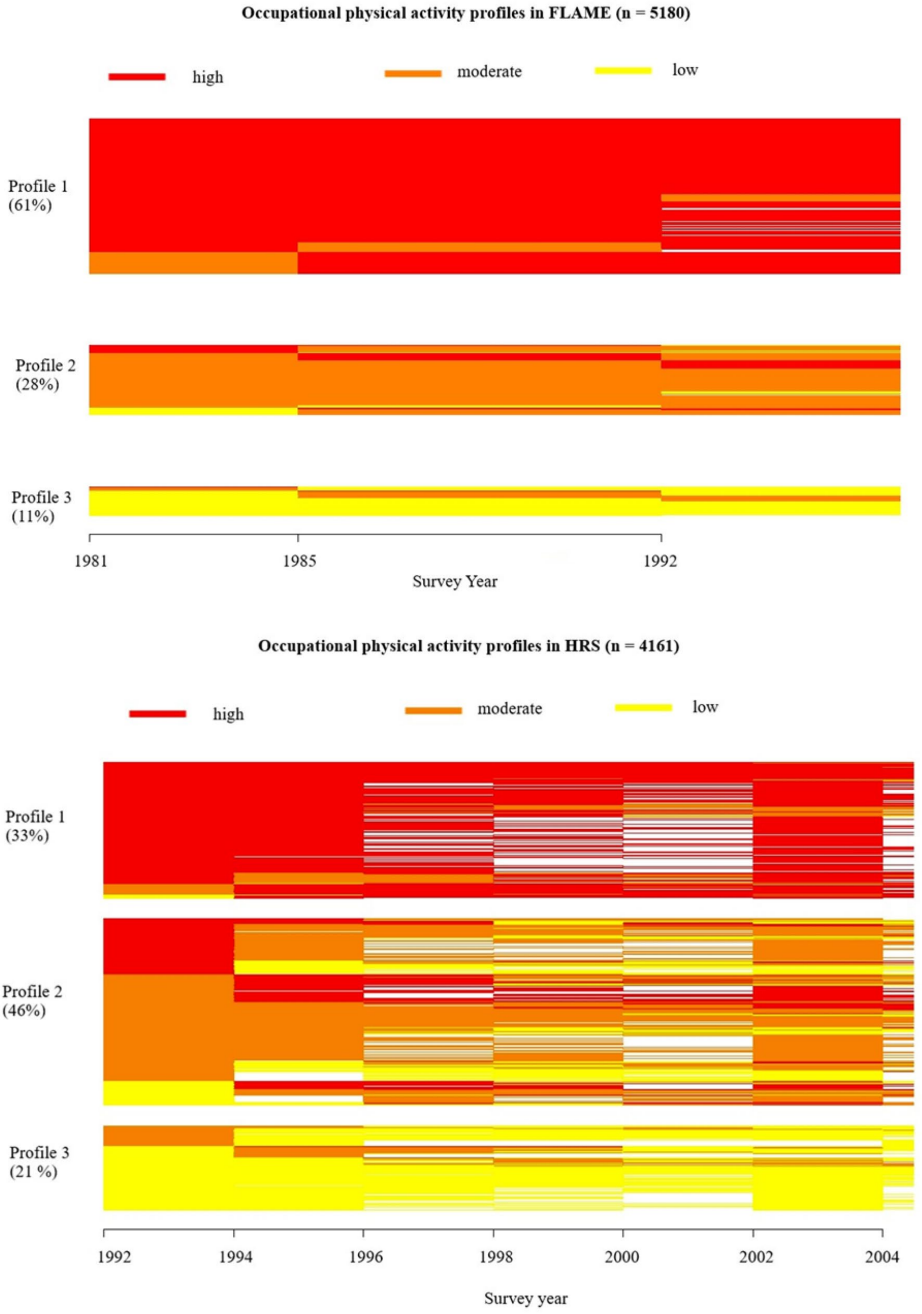
Horizontal line plots of profiles of occupational physical activity in FLAME from 1981 to 1997 (n = 5108) and in HRS from 1992 to 2006 (n = 4161). Each horizontal line represents a participant, and the colors differentiate between levels of occupational physical activity. White color indicates missing data. Profile 1 = High-persistent, Profile 2 = Moderate-fluctuating, Profile 3 = Low-persistent

In both study cohorts, three distinct OPA profiles were identified, within which participants reported similar patterns of OPA during the follow-up (Fig. 1).

The most common profile (61%) among FLAME participants was the high-persistent profile, in which the majority reported a high level of OPA at each time point. No participants in this profile reported low OPA at any time point, however, some fluctuated between a high and a moderate level during the follow-up. In HRS a third of the respondents belonged to the high-persistent profile.

The second-largest profile (28%) in FLAME was the moderate-fluctuating profile which was characterized by a high likelihood of reporting moderate OPA at each time point, yet some fluctuation between the levels of OPA occurred in this profile. In HRS the moderate-fluctuating profile was the largest (46%) in which a high fluctuation between levels of OPA occurred.

A small proportion of FLAME respondents (11%) were classified in the low-persistent profile, which was characterized by a high likelihood of reporting a low level of OPA throughout the follow-up. In HRS, approximately one-fifth of the respondents were classified into the low-persistent -profile.

### Modelling of the distal outcomes

The estimated RRs with 95% CIs for mobility limitations according to OPA profiles are presented in Table 2. Overall, in FLAME the absolute risk for mobility limitations was higher for those in the high-persistent profile (872/1446, 60%) and in the moderate-fluctuating profile (372/769, 48%) compared to those in the low-persistent profile (146/329, 44%). The RR for mobility limitations was 1.32 (95% CI 1.16–1.50) for high-persistent OPA in age and sex adjusted model for FLAME participants. Further adjustment for education, life-style factors, BMI and chronic conditions attenuated the RR (RR 1.19, 95% CI 1.04–1.35). The risk estimates for moderate-fluctuating OPA were not statistically significant.

**Table 2:** Risk ratios (RR) and their 95% confidence intervals (CI) for mobility limitations in old age according to occupational physical activity profile (OPA profile) membership in FLAME (n = 2544) and in HRS (n = 2126) cohorts

OPA profile	n	Cases n (%)	Model 1^a^ RR (95% CI)	Model 2^b^ RR (95% CI)
FLAME			n = 2544	n = 2459^c^
Low-persistent	329	146 (44)	ref	ref
Moderate-fluctuating	769	372 (48)	1.12 (0.98–1.29)	1.08 (0.95–1.24)
High-persistent	1446	872 (60)	1.32 (1.16–1.50)	1.19 (1.04–1.35)
HRS			n = 2126	n = 2116^d^
Low-persistent	446	228 (51)	ref	ref
Moderate-fluctuating	968	599 (62)	1.20 (1.09–1.33)	1.13 (1.03–1.25)
High-persistent	712	440 (62)	1.22 (1.10–1.35)	1.12 (1.01–1.25)
Pooled estimates				
Low-persistent			ref	ref
Moderate-fluctuating			1.18 (1.08–1.27)	1.11 (1.03–1.21)^e^
High-persistent			1.26 (1.16–1.36)	1.15 (1.06–1.25)^f^

^a^Adjusted for age and sex

^b^Adjusted for Model 1 + education level, leisure-time physical activity, smoking, alcohol consumption, body-mass index, chronic conditions (cardiovascular disease, respiratory disease, musculoskeletal disease, diabetes) and accidental injuries

^c^n = 85 excluded due to missing values.

^d^n = 10 excluded due to missing values.

^e^(I^2^ < 0.01%, p = 0.62),

^f^(I^2^ < 0.01%, p = 0.53)

In HRS, mobility limitations occurred more often among those in the high-persistent profile (440/712, 62%) and in the moderate-fluctuating profile (599/968, 62%) as compared to those in the low-persistent profile (228/446, 51%). RR of mobility limitation for both high-persistent and moderate-fluctuating OPA in age and sex adjusted model remained statistically significant in the fully adjusted model with RR of 1.12 (95% CI 1.01–1.25) for the high-persistent and RR of 1.13 (95% CI, 1.03–1.25) for the moderate-fluctuating profile.

Pooled estimates for mobility limitations were statistically significant for both the high-persistent- (adjusted RR 1.15, 95% CI 1.06–1.25) and the moderate-fluctuating- (adjusted RR 1.11, 95% CI 1.03–1.21) profiles. I^2^ statistics showed no between-study heterogeneity (Table 2).

The respective RRs for ADL-disability are presented in Table 3. In FLAME ADL-disability was higher among those in the high-persistent profile (409/1488, 28%) compared with the moderate-fluctuating profile (159/775, 21%), and the low-persistent profile (60/333, 18%). The sex- and age-adjusted RR was statistically significant for the high-persistent profile (1.60, 95% CI 1.26–2.04) but the risk was attenuated and did not remain statistically significant in the fully adjusted model (RR 1.28, 95% CI 0.99–1.66).

**Table 3:** Risk ratios (RRs) and their 95% confidence intervals (CIs) for ADL-disability in old age according to occupational physical activity profile (OPA profile) membership in FLAME (n = 2596) and in HRS (n = 1299) cohorts

OPA profile	n	Cases n (%)	Model 1^a^ RR (95% CI)	Model 2^b^ RR (95% CI)
FLAME			n = 2596	n = 2513^c^
Low-persistent	333	60 (18)	ref	ref
Moderate-fluctuating	775	159 (21)	1.17 (0.90–1.53)	1.07 (0.82–1.40)
High-persistent	1488	409 (28)	1.60 (1.26–2.04)	1.28 (0.99–1.66)
HRS			n = 1299	n = 1295^d^
Low-persistent	224	68 (30)	ref	
Moderate-fluctuating	626	165 (26)	0.88 (0.69–1.11)	0.84 (0.66–1.07)
High-persistent	449	152 (34)	1.13 (0.89–1.43)	1.10 (0.86–1.42)
Pooled estimates				
Low-persistent			ref	ref
Moderate-fluctuating			1.01 (0.76–1.34)	0.94 (0.74–1.19)^e^
High-persistent			1.34 (0.95–1.89)	1.19 (0.99–1.42)^f^

Note. ADL = Actitivities of daily living

^a^Adjusted for age and sex

^b^Adjusted for Model 1 + education level, leisure time physical activity, alcohol intake, smoking, body mass index, chronic conditions (cardiovascular disease, respiratory disease, musculoskeletal disease, diabetes) and accidental injuries

^c^n = 83 excluded due to missing values

^d^n = 4 excluded due to missing values

^e^(I^2^ = 43.0%, p = 0.19)

^f^(I^2^ < 0.01%, p = 0.41)

In HRS absolute risk for ADL-disability was the highest among those in the high-persistent profile (153/449, 34%), followed by those in the low-persistent profile (68/224, 30%), and the lowest for those in the moderate-fluctuating profile (165/626, 26%). The adjusted RRs were not statistically significant for either group.

The fully adjusted pooled estimates were not statistically significant for either group, and we observed moderate heterogeneity between the studies according to I^2^ statistics (43% for the moderate-fluctuating profile, and 41% for the high-persistent profile).

### Death as a competing risk for ADL-disability

In FLAME 2252 respondents (54%) experienced the competing outcome (Table 4). The adjusted risk was 18% higher for those in the high-persistent than among those in the low-persistent OPA profile (adjusted RR 1.18, 95% CI 1.06–1.31). In HRS 1645 respondents (63%) experienced the outcome. We detected a 10% lower risk for those in the moderate-fluctuating than among those in low-persistent OPA profile (adjusted RR 0.90, 95% CI 0.84–0.97). There was no difference in the risk of competing outcome among those in high-persistent and those in low-persistent OPA profile.

**Table 4:** Risk ratios (RRs) and their 95% confidence intervals (CIs) for ADL-disability or death according to occupational physical activity profile membership in FLAME (n = 4156) and in HRS (n = 2559) cohorts

OPA profile	n	Cases n (%)	Model 1^a^ RR (95% CI)	Model 2^b^ RR (95% CI)
FLAME			n = 4156	n = 4006^c^
Low-persistent	495	222 (45)	ref	ref
Moderate-fluctuating	1176	560 (48)	1.10 (0.98–1.22)	1.04 (0.93–1.16)
High-persistent	2485	1406 (57)	1.35 (1.23–1.50)	1.18 (1.06–1.31)
HRS			n = 2559	n = 2547^d^
Low-persistent	478	322 (67)	ref	
Moderate-fluctuating	1180	719 (61)	0.93 (0.86–1.00)	0.90 (0.84–0.97)
High-persistent	901	604 (67)	1.02 (0.94–1.10)	0.99 (0.91–1.07)
Pooled estimates				
Low-persistent			ref	ref
Moderate-fluctuating			1.00 (0.85–1.18)	0.96 (0.84–1.11)^e^
High-persistent			1.17 (0.88–1.55)	1.07 (0.90–1.28)^f^

Note. ADL = Actitivities of daily living

^a^Adjusted for age and sex

^b^Adjusted for Model 1 + education level, leisure time physical activity, alcohol intake, smoking, body mass index, chronic conditions (cardiovascular disease, respiratory disease, musculoskeletal disease, diabetes) and accidental injuries

^c^n = 150 excluded due to missing values

^d^n = 12 excluded due to missing values

^e^(I^2^ = 76.8%, p = 0.04)

^f^(I^2^ = 85.5%, p = 0.01)

The fully adjusted pooled estimates were not statistically significant for either group, and we observed considerable heterogeneity between the studies according to I^2^ statistics (77% for the moderate-fluctuating profile, and 86% for the high-persistent profile).

### Attrition analysis

Slightly less than a third of the study sample died during the follow-up in both cohorts. The included sample was younger, had higher education, lower BMI, less chronic conditions, and detrimental lifestyles as compared to those who died in both cohorts (Table S2). In FLAME, they belonged more often to the high-persistent OPA profile than those who had the outcome information, but this was not evident in HRS. In FLAME similar, yet not as strong differences were found between the included participants and those participants who dropped out. In contrast, in HRS those who were lost from follow-up were overall younger and in better health than those who were included. Also, they more often belonged to the low-persistent OPA profile than the included respondents.

## Discussion

In this population-based cohort study of Finnish and American ageing workers we identified three distinct profiles of OPA among both cohorts, namely high-persistent, moderate-fluctuating, and low-persistent. For the majority, OPA remained rather stable during these last years of an occupationally active life—more so among those reporting either high or low levels of OPA at baseline, whereas more within-person changes occurred in the moderate-fluctuating profile. Furthermore, the study findings indicate that moderate and high OPA levels are associated with an increased risk of mobility limitations at old age.

### OPA profiles

To address contextual factors, e.g., cohort effects as well as potential cultural effects arising from different geographical locations, we studied the developmental profiles of OPA among two different cohorts of aging workers. FLAME covered a wide range of occupations in the municipal sector in Finland, and workers' exposure to OPA was monitored from the beginning of the 80 s to the beginning of the 90 s. HRS on the other hand comprised American general working population whose exposure to OPA was monitored from the beginning of the 90 s to the mid-2000s. Analyzing longitudinal data on OPA among two cohorts, from two different countries, and two different decades, we identified three distinct OPA profiles.

The results showed that the exposure to high OPA likely keeps accumulating during the last years of occupationally active life, as those reporting high OPA at baseline were likely to keep reporting it throughout the follow-up. Similarly, the perceived physical requirements of work remained rather constant for people reporting low OPA at baseline. This is logical—in physically light office work, the physical demands of work do not often change, and at an older age, it is unlikely to change the profession to a physically more demanding one. The fit indices (Table S1) showed that employees whose physical job demands remained unchanged, either at a high or a low level, were clearly separated into distinct profiles. However, our analyses also revealed that OPA does not remain constant for all aging workers, i.e., in FLAME altogether 28% of the study participants, and in HRS 46% were classified into a moderate- fluctuating profile—they were most likely the ones who reported moderate OPA at the baseline.

In very few studies, the longitudinal patterns of physical work demands among aging employees have been investigated. Therefore, we lack data to compare our results to. Swedish researchers showed in their recent study (Åkerstedt et al. 2019) among an occupationally active cohort that perceived physical work demands slightly decreased over an eight-year follow-up. The decrease was steeper among blue-collar workers as compared to white-collar workers, and smaller in the oldest (57–68 years) as compared to the younger age groups. Prior cross-sectional evidence has also shown a linear declining trend for physically demanding work by age (Aittomäki et al. 2005), yet, to our knowledge, in previous studies a person-centered approach has not been used.

### OPA profile as a risk factor of mobility limitations and ADL-disability

With aging, the physical work capacity typically declines as the body’s systems become less able to repair damage caused by accumulated exposures (Ilmarinen 2002). The period from mid-working life to retirement can be considered *a sensitive period*—a limited time window when exposure has a stronger effect on the structure or function of organs, tissues, or body systems (D Kuh et al. 2003). Our findings showed that respondents exposed to high consistent OPA more likely experienced difficulties with mobility tasks in old age. A consistently higher risk of mobility limitations was found for people classified into the high-persistent OPA profile as compared to people in the low-persistent OPA profile. The risk was higher also for those in the moderate-fluctuating profile, although in FLAME the association was not statistically significant. Our results accord with previous findings which have shown that physically demanding work is associated with worse physical performance in late life (Hinrichs et al. 2014; Leino-Arjas et al. 2004; Mänty et al. 2014; Russo et al. 2006; Sabbath et al. 2013). The use of repeated measurements over more than a decade as well as the person-centered analytic approach allowed us to also investigate the accumulation of the exposure and its consequences among ageing workers.

Physical work demands are risk factors for the development of musculoskeletal disorders (Kumar 2001). Diminishing muscle function capacities is a likely mechanism through which OPA may result in a reduced mobility many years later (Nygård et al. 1994). Mobility limitations may eventually compromise one’s ability to perform daily activities independently. OPA has been shown to predict ADL-disability (Mc Carthy et al. 2013; Prakash et al. 2017; Sterud 2014). In contrast, we did not find statistically significant associations between profiles of OPA and subsequent ADL-disability. In both cohorts the high-persistent OPA profile had higher absolute risk of ADL-disability, but after adjusting for confounders this association disappeared. In accordance, in a previous Swedish study, no association between high OPA and ADL-disability after controlling for demographic and health-related factors was found (Rydwik et al. 2013). A possible explanation to this null finding is selective mortality, which changes the composition of aging cohorts over time, and as a result the cohorts appear “healthier, wealthier, more educated, and generally better off than they would in the absence of the selection process” (Zajacova and Burgard 2013). A considerably high proportion of the study sample died during the study period before censoring for ADL-disability, likely attenuating the calculated risk estimates in our study. In our sensitivity analysis, where we incorporated death as a competing risk for disability, the results showed that in FLAME, the adjusted risk was 18% higher for members in the high-persistent OPA profile than to members of the low-persistent profile. In HRS, on the other hand, the risk did not differ between low-persistent and high-persistent OPA-profile. Interestingly, the risk was 10% lower for members in the moderate-fluctuating profile as compared to members of the low-persistent profile, which is in line with the previous findings by Rydwik et al. (2013), in which moderate levels of OPA during midlife were found to be associated with a decreased risk of disability. However when stratified by the occupational class, the higher risk remained among white-collar but not among blue-collar workers (Rydwik et al. 2013).

Selective differences between people in the different OPA profiles may have contributed to the risk estimates. By controlling the level of education, we aimed to control for the confounding that arises from different socioeconomic statuses (Cuthbertson, Moore, and Evenson 2023). We did not adjust the models for occupational status (e.g., blue- vs. white-collar workers) as the level of OPA can be thought to be a direct outcome of occupational status, thus controlling for it would have led to over-adjustment. We however adjusted our models with several lifestyle factors and chronic health conditions. The risk estimates differed markedly between the minimally and fully adjusted models verifying the confounding role of these factors in the associations. We cannot however rule out the possibility of residual confounding.

### Strengths and limitations

The major strength of this study is the use of longitudinal data on the exposure to OPA and a person-centered analyses method to show different profiles of OPA, which allows to investigate the variation within occupations or within individuals across time (Andrasfay, Pebley, and Goldman 2021). Another strength is the use of data from two well-established cohort studies that have followed people for nearly three decades from midlife until old age. Compared to other observational studies, prospective cohort studies with such a long follow-up deliver stronger evidence of the association. The data on OPA was collected across two different decades in two different cultural settings, which further strengthens our study.

The potential limitation of this study is the use of self-reported OPA assessments. Objectively measured information would be more accurate, however, when studying the long-term consequences, the use of longitudinal data based on employee surveys or interviews on self-assessed OPA is nevertheless justified. The subjective assessment not only postulates the level of the physical demands of the job, but also describes the change in the relationship between the respondents' physical capacity and the physical demands of the job. When physical capacity declines, for example due to aging changes, a constant, objectively measured load level may require a relatively greater effort from the employee, in which case the perceived workload may be higher. In an earlier study, researchers showed that the self-reported physical workload increased when approaching retirement age, even though objectively assessed, the physical demands of the work remained the same over a twelve-year follow-up (Nygård et al. 1997).

It is also possible that there are sex-based differences in the long-term consequences of exposure to OPA. We did not find effect modification by sex as assessed by the magnitude and the statistical significance of the cross-product term between OPA profile and sex with respect to the outcomes. In previous studies, however, different associations between OPA and mortality have been found for male and female workers (Coenen et al. 2018) and it has been suggested that there might be sex differences e.g., in physiological responses to OPA behind this finding (Hinrichs et al. 2014). Moreover, men and women may perceive and report the intensity of OPA differently, which should be considered in future studies.

It needs also to be noted that attrition was considerably high. It is thus possible that our results were affected by selective loss-to follow-up. In FLAME the dropouts were older and had more chronic conditions than those who were included which likely attenuated the risk estimates. On the other hand, in HRS those who were dropped out were younger and healthier than those who were included, which may partly explain the contradicting results between the two cohorts with regards to the ADL-disability.

## Conclusions

For workers reporting high or low levels of OPA at midlife, the physical demands of work likely persist until their last occupationally active years, whereas people reporting moderate level OPA at midlife have high fluctuation in their exposure level.

Our results contribute to the wide evidence base of the long-reaching adverse consequences of OPA.

The results of this study increase the understanding of how OPA during late working life develop and influence functional ability in later life. According to our results high-persistent OPA increases the risk of mobility limitations at old age suggesting that mitigating the exposure to physical work demands during midlife to retirement might be conducive to healthy aging. The potential benefits of this research to society include a better functioning aging population as the results provide evidence for planning effective and timely organizational and policy interventions targeted to those in physically demanding occupations. The results can also be used at an individual level to promote health and functioning in later life.

### Supplementary Information

Below is the link to the electronic supplementary material.

Supplementary file 1: (DOCX 140 KB)

## Data Availability

The data to support the findings of this study are available upon reasonable request from the corresponding author. Restrictions apply to the availability of the data, which were used under license for the current study.
