# Supplementary material for: Longitudinal profiles of occupational physical activity during late midlife and their association with functional limitations at old age: a multi-cohort study
Source: Int Arch Occup Environ Health. 2023 Jul 29;96(9):1245–56. doi: 10.1007/s00420-023-02003-5 (PMC10560154; doi:10.1007/s00420-023-02003-5)
Supplement: Supplementary file 1 — (DOCX 140 KB) [file 420_2023_2003_MOESM1_ESM.docx]

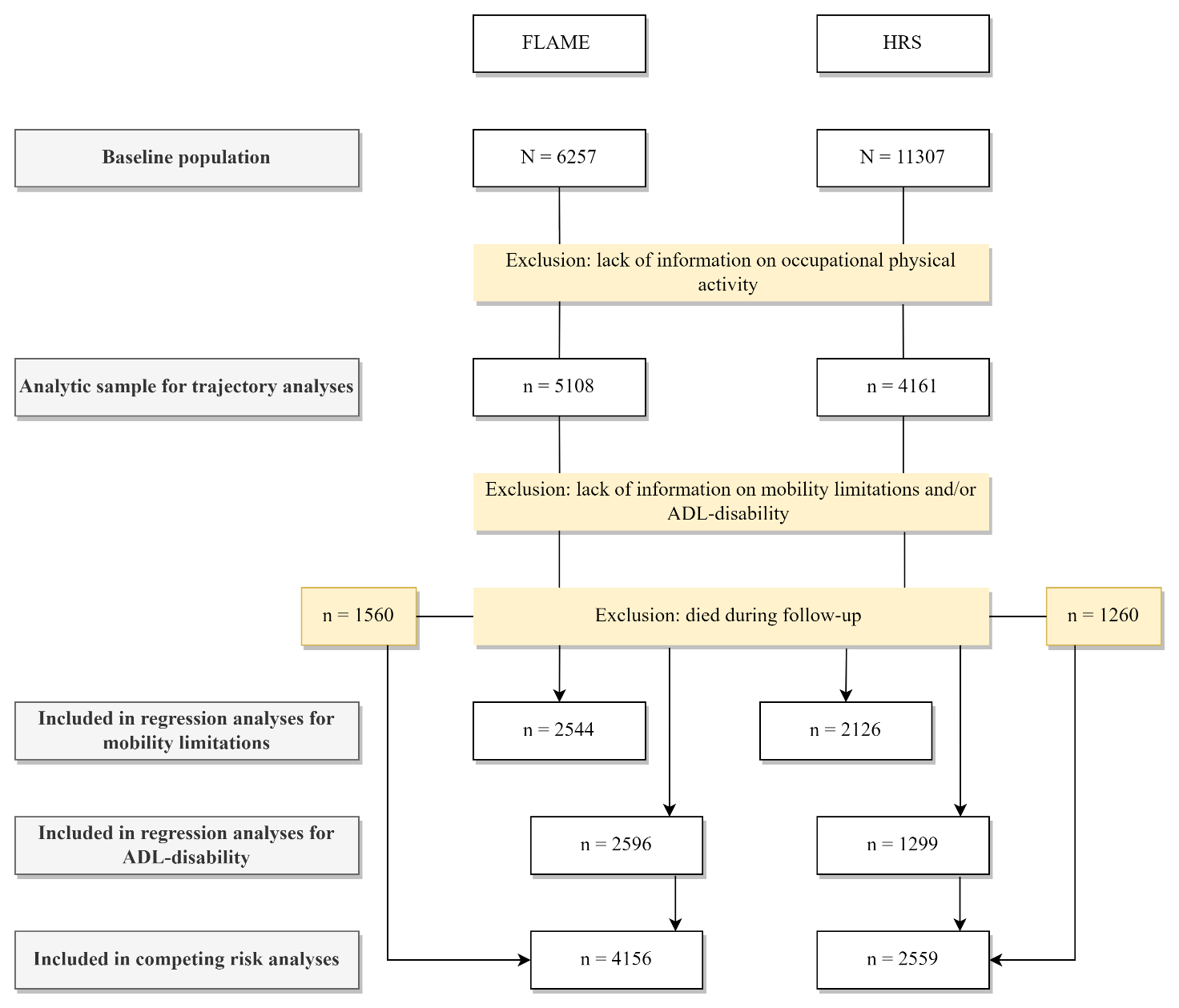


**Fig 1** Flowchart of Finnish Longitudinal Study of Ageing Municipality Employees (FLAME) and Health and Retirement (HRS) respondents included in the study

**Supplementary Table 1.** Model fit indices of latent class growth analysis: occupational physical activity profiles in FLAME cohort during 1981 to 1992 (n = 5108) and in HRS cohort during 1992 to 2004 (n = 4161)

| Number of latent classes | Polynomial shape of the fitted curve | AIC | BIC | Class proportions | Entropy | Estimated posterior probabilities |
| --- | --- | --- | --- | --- | --- | --- |
| FLAME |  |  |  |  |  |  |
| 1 | i,s,q | 25362.3 | 25388.5 | 1 | na | 1 |
| 2 | i,s,q | 21052.1 | 21104.4 | 0.32/0.68 | 0.83 | 0.95/0.94 |
| **3** | **i,s,q** | **20069.3** | **20147.7** | **0.60/0.28/0.11** | **0.81** | **0.93/0.89/0.88** |
| 4* | i,s,q |  |  |  |  |  |
| 4 | i,s | 20095.1 | 20173.6 | 0.07/0.10/0.28/0.41 | 0.64 | 0.78/0.92/0.84/0.75 |
| 5* | i,s,q |  |  |  |  |  |
| 5 | i,s | 20085.0 | 20183.1 | 0.00/0.54/0.27/0.09/0.10 | 0.70 | 0.76/0.78/0.80/0.77/0.93 |
|  |  |  |  |  |  |  |
|  |  |  |  |  |  |  |
| HRS |  |  |  |  |  |  |
| 1 | i,s,q | 26157.9 | 26183.3 | 1 | na | 1 |
| 2 | i,s,q | 23787.7 | 23838.4 | 0.51/0.49 | 0.68 | 0.94/0.87 |
| **3** | **i,s,q** | **23487.5** | **23563.5** | **0.44/0.23/0.33** | **0.57** | **0.73/0.79/0.87** |
| 3 | i,s | 23495.9 | 23552.9 | 0.45/0.22/0.33 | 0.58 | 0.72/0.78/.0.79 |
| 3 | i,s,q,c | 23491.4 | 23586.4 | 0.44/0.23/0.33 | 0.57 |  |
| 4* | i,s,q |  |  |  |  |  |
| 4 | i,s | 23474.2 | 23550.2 | 0.33/0.22/0.44/0.01 | 0.61 | 0.85/0.79/0.80/0.59 |
| 5* | i,s,q |  |  |  |  |  |
| 5* | i,s |  |  |  |  |  |
| **3** | **class 1: i,s,q**  **class 2: i,s**  **class 3: i,s** | **23484.8** | **23548.2** | **0.46/0.21/0.33** | **0.58** | **0.72/0.82/0.87** |

*Note.* AIC= Akaike Information Criterion, BIC= Bayesian Information Criterion.*Model was not identified due to singularity.

**supplementary Table 2.** Differences between participants who were included into the regression analyses, died, or dropped out. Summary statistics calculated among those who were included into the trajectory analysis (n = 5108 in FLAME, n = 4161 in HRS,)

|  | **FLAME** | | | | **HRS** | | | |
| --- | --- | --- | --- | --- | --- | --- | --- | --- |
|  | Included  (n = 2596) | Died during follow-up  (n =1560) | Dropped out  (n = 952) | p-value for difference | Included  (n = 1299) | Died during follow-up  (n =1260) | Dropped out  (n = 1602) | p-value for difference |
| Age, years, mean (SD) | 49.6 (3.4) | 51.5 (3.6) | 50.3 (3.6) | <0.001 | 55.2 (3.5) | 56.8 (4.2) | 54.6 (3.3) | <0.001 |
| Women, % | 63 | 40 | 65 | <0.001 | 58 | 35 | 46 | <0.001 |
| Leisure-time physical activity, % |  |  |  | <0.001 |  |  |  | <0.001 |
| Low | 21 | 23 | 22 |  | 21 | 22 | 18 |  |
| Moderate | 27 | 33 | 29 |  | 22 | 20 | 18 |  |
| High | 53 | 44 | 48 |  | 57 | 57 | 64 |  |
| Education, % |  |  |  | <0.001 |  |  |  | <0.001 |
| low | 25 | 41 | 35 |  | 24 | 32 | 16 |  |
| intermediate | 62 | 52 | 55 |  | 45 | 39 | 42 |  |
| high | 13 | 7 | 9 |  | 32 | 29 | 42 |  |
| Body mass index |  |  |  | <0.001 |  |  |  | <0.001 |
| <25kg/m^2^ | 51 | 41 | 46 |  | 34 | 30 | 42 |  |
| 25-30 kg/m^2^ | 42 | 47 | 46 |  | 41 | 44 | 42 |  |
| >30 kg/m^2^ | 7 | 12 | 8 |  | 25 | 26 | 15 |  |
| Alcohol consumption, % |  |  |  | <0.001 |  |  |  | <0.001 |
| Abstinent | 74 | 59 | 74 |  | 37 | 37 | 30 |  |
| Moderate | 19 | 24 | 19 |  | 50 | 45 | 55 |  |
| High | 7 | 17 | 8 |  | 14 | 18 | 16 |  |
| Smoking, % |  |  |  | <0.001 |  |  |  | <0.001 |
| Never | 65 | 41 | 59 |  | 42 | 25 | 44 |  |
| Former | 24 | 29 | 25 |  | 38 | 37 | 40 |  |
| Current | 11 | 31 | 16 |  | 21 | 37 | 16 |  |
| Chronic conditions, % |  |  |  |  |  |  |  |  |
| Respiratory disease | 10 | 15 | 13 | <0.001 | 9 | 10 | 8 | <0.001 |
| Cardiovascular disease | 17 | 30 | 19 | <0.001 | 9 | 13 | 6 | <0.001 |
| Musculo-skeletal disease | 31 | 37 | 32 | <0.001 | 38 | 36 | 26 | <0.001 |
| Diabetes | 2 | 3 | 2 | <0.001 | 6 | 12 | 4 | 0.002 |
| Accidental injury | 10 | 16 | 11 | <0.001 | 13 | 14 | 12 | <0.001 |
| OPA profile, % |  |  |  | <0.001 |  |  |  | <0.001 |
| low-persistent | 13 | 10 | 8 |  | 17 | 20 | 24 |  |
| moderate-fluctuating | 30 | 26 | 24 |  | 48 | 44 | 46 |  |
| high-persistent | 57 | 64 | 68 |  | 35 | 36 | 30 |  |
